# Risikomanagement in der Triage ambulanter Notfallpatienten: Manchester Triage System und CEReCo-blue als Instrument zur risikoarmen Patientensteuerung in integrierten Notfallzentren

**DOI:** 10.1007/s00063-021-00853-w

**Published:** 2021-08-27

**Authors:** Dirk Pabst, Jonas Schibensky, David Fistera, Joachim Riße, Clemens Kill, Carola Holzner

**Affiliations:** Zentrum für Notfallmedizin, Universitätsmedizin Essen, Hufelandstr. 55, 45147 Essen, Deutschland

**Keywords:** Ersteinschätzung, Notfallmedizin, Notfalltresen, Ambulante Patienten, Stationäre Aufnahme, Primary assessment, Emergency medicine, Emergency counter, Outpatients, Hospital admission

## Abstract

**Hintergrund:**

Zur frühzeitigen Entscheidung in zukünftigen „Integrierten Notfallzentren“, ob eine ambulante oder innerklinische Versorgung indiziert ist, wäre es hilfreich, ein System zu haben, mit dem die Identifizierung von Patienten mit ambulanter Behandlungsindikation möglich ist. In dieser Studie untersuchten wir, ob das Manchester Triage System (MTS) dafür geeignet ist, Patienten zu erkennen, die sicher der ambulanten medizinischen Versorgung zugeteilt werden können.

**Methode:**

Notaufnahmepatienten der „blauen“ MTS-Dringlichkeitsstufe wurden auf den Endpunkt „stationäre Aufnahme“ untersucht und mit der nächsthöheren MTS-Kategorie „grün“ verglichen. In einem zweiten Schritt wurde die „blaue“ Dringlichkeitsstufe auf die häufigsten gemeinsamen Kriterien untersucht, die zur stationären Aufnahme führten.

**Ergebnisse:**

Nach Ausschluss von Patienten, die durch den Rettungsdienst oder nach vorherigem Arztbesuch vorstellig wurden, war die Rate der stationären Aufnahmen in der blauen Dringlichkeitsstufe signifikant niedriger als in der grünen Kategorie (10,8 % vs. 29,0 %). Die Rate konnte durch die Etablierung einer Untergruppe mit den zusätzlichen Ausschlusskriterien chronische Erkrankung und Wiedervorstellung nach vorheriger stationärer Behandlung auf 0,9 % gesenkt werden. (CEReCo-blue-Gruppe: **C**hronic Disorder (C), **E**mergency Medical Service (E), **Re**admission (R), Prior Medical **Co**nsultation (Co)).

**Schlussfolgerung:**

Die blaue MTS-Dringlichkeitsstufe scheint zur Selektion von Patienten mit ambulanter Behandlungsindikation nicht geeignet zu sein. Wir schlagen die Einführung einer Untergruppe, der sog. CEReCo-blue-Gruppe vor, die für die Selektion dieser Patientengruppe hilfreich sein könnte.

## Abstract (en)

**Background:**

So-called “integrated emergency centers (IEC)” are going to be implemented in German hospitals and will be the first contact point in the emergency care setting. For early decision-making whether outpatient care or inpatient admission in the emergency department is indicated, it would be helpful to have a system to identify patients for outpatient treatment. In this study, we investigated whether the Manchester Triage System (MTS) is suitable for identifying patients who can be safely referred to emergency room patients to outpatient care.

**Methods:**

Patients in the emergency department of the “blue” MTS level were examined for the endpoint inpatient admission and were compared with the next higher MTS category “green”. In a second step, the “blue” MTS category was examined for the most common criteria leading to inpatient admission.

**Results:**

After excluding patients who were presented by the emergency medical services (EMS) or after prior medical consultation, the rate of inpatient admissions in the blue MTS category was significantly lower than in the green category (10.8% vs 29.0%). The rate could be reduced to 0.9% by establishing a subgroup with the additional exclusion criteria chronic disorder and readmission after prior inpatient treatment (CEReCo-blue group: **C**hronic Disorder (C), **E**mergency Medical Service (E), **Re**admission (R), Prior Medical **Co**nsultation (Co)).

**Conclusion:**

The blue MTS category does not appear to be suitable for the selection of patients with indication for outpatient treatment. We propose the introduction of a subgroup, the so-called CEReCo-blue group, which could be helpful for the selection of this patient group.

Weltweit werden in Notaufnahmen steigende Patientenzahlen festgestellt [2]. Daher scheint es notwendig, Strukturen zu schaffen, die ein zu hohes Patientenaufkommen verhindern und dabei weiterhin eine qualitativ hochwertige Patientenversorgung gewährleisten [2]. Es wird aktuell diskutiert, wie durch frühes Erkennen von Patienten, die keiner weiteren stationären Behandlung bedürfen und einer ambulanten Behandlung beim niedergelassenen Arzt oder kassenärztlichen Vertretungsarzt (KV-Arzt) zugeteilt werden könnten, die Anzahl der Patienten in den Notaufnahmen reduziert werden könnte. Durch „Gatekeeping“ an einem zentralen Tresen der Notfallzentren könnte eine Überfüllung der Notaufnahmen durch Verringerung der Anzahl nicht dringender Fälle reduziert werden [2, 14].

Der Gesetzgeber in Deutschland strebt die Einführung integrierter Notfall- und Koordinationszentren an, die durch eine Kooperation stationärer und ambulanter Dienste mit geteilter Finanzierung betrieben werden. Es sollen Instrumente implementiert werden, um Patienten zu identifizieren, die sicher an ambulante Dienste verwiesen werden könnten [5]. Hierfür könnten existierende Triage-Systeme eingesetzt werden [4]. Die vier international etabliertesten Triage-Systeme sind die Australasian Triage Scale (ATS, Australien/Neuseeland), die Canadian Triage and Acuity Scale (CTAS, Kanada), der Emergency Severity Index (ESI, USA) und die Manchester Triage Scale (MTS, Großbritannien) [16, 17]. Jedes dieser Systeme verfügt über eine 5‑stufige Skala, die Fälle mit dem höchsten Schweregrad 1 bis zum niedrigsten Schweregrad 5 kategorisiert [1, 7, 9, 16, 17]. In Deutschland ist das MTS das häufigste System und wird seit 2004 offiziell eingesetzt [7]. Das MTS basiert auf der Registrierung führender Symptome, die 52 verschiedenen Präsentationsdiagrammen zugeordnet werden können. Während des weiteren Verfahrens werden die Fälle in Bezug auf fünf Schweregrade priorisiert, anhand derer die Dringlichkeit des Arztkontakts vorgegeben wird: rot = sofortige Behandlung (0 min), orange = sehr dringend (10 min), gelb = dringend (30 min), grün = normal (90 min), blau = nicht dringend (120 min) [9, 17]. Bisher hat sich kein System als zuverlässig gezeigt, Patienten zu identifizieren, die sicher an die ambulante Versorgung verwiesen werden können [14]. In einer prospektiven Studie untersuchten Slagman et al. [12], ob das MTS dazu geeignet ist, „non-urgent“ Patienten mit ambulanter Behandlungsindikation zu erkennen. Dafür wurden Patienten der blau und grünen Dringlichkeitsstufe als „non-urgent“ Patienten definiert, die restlichen Dringlichkeitsstufen wurden als „urgent“ Patienten mit dringender Behandlungsindikation eingestuft. Die Studie zeigte eine stationäre Aufnahmerate bei den „non-urgent“ Patienten von immerhin 29,6 % und konnte keinen signifikanten Unterschied in der Kurz- und Langzeitsterblichkeit zwischen den „non-urgent“ und den „urgent“ Patienten zeigen [12], sodass die Autoren daraus schlossen, dass das MTS für die Selektion von Patienten, die an ambulante Behandlungseinrichtungen verwiesen werden könnten, ungeeignet ist. Eine gesonderte Untersuchung der blauen Dringlichkeitsstufe wurde in der Studie jedoch nicht vorgenommen.

Wir sind in unserer Studie davon ausgegangen, dass die blaue Dringlichkeitsstufe des MTS oder eine weitere Untergruppe der blau triagierten Patienten dabei helfen könnte, frühzeitig Patienten zu erkennen, die sicher dem ambulanten Versorgungssystem zugeordnet werden könnten. Ziel der vorliegenden Studie war daher, das Manchester Triage System anhand der blauen Dringlichkeitsstufe auf die Zuverlässigkeit zu untersuchen, Patienten mit ambulanter Behandlungsindikation zu erkennen.

## Methoden

In diese monozentrische Studie wurden retrospektiv alle erwachsenen (Alter ≥18 Jahre), nichttraumatologischen, nichtneurologischen Patienten eingeschlossen, die zwischen Januar 2019 und Dezember 2019 in der zentralen Notaufnahme des Universitätsklinikums Essen vorstellig wurden. Patienten, die sich während des Studienzeitraums häufiger vorstellten, wurden nur einmalig in die Studie eingeschlossen. Patienten wurden ausgeschlossen, wenn sie über den Rettungsdienst aufgenommen wurden oder mit der gleichen Beschwerdesymptomatik bereits vorher einen Arzt konsultiert hatten.

Die Patienten der blauen Dringlichkeitsstufe wurden zunächst mit den Patienten der nächst höheren Dringlichkeitsstufe „grün“ bezüglich einer stationären Aufnahme verglichen. In einem weiteren Schritt untersuchten wir die häufigsten gemeinsamen Merkmale von Patienten der blauen Kategorie, die stationär aufgenommen wurden. Des Weiteren wurden Kriterien für eine „gerechtfertigte stationäre Aufnahme“ definiert (Tab. 1).

**Table 1:** 

Eine stationäre Aufnahme eines Patienten wurde als „gerechtfertigte stationäre Aufnahme“ definiert, wenn mindestens eines der folgenden Kriterien zutraf
*Notfallintervention:* Koronarangiographie, Endoskopie (ÖGD, Koloskopie, ERCP), Notfalloperation
*Relevante Diagnose in der Computertomographie (CT) mit direkter therapeutischer Konsequenz*
*Aufnahme auf eine Intensivstation (ICU) oder Intermediate Care Unit (IMC)*
*Zeichen einer frischen Myokardischämie, tachykarde oder bradykarde Rhythmusstörungen im EKG*
*Veränderte Laborparameter in der „point-of-care diagnostics“ (POCD) oder im Zentrallabor* erhöhtes Troponin, erhöhte D‑Dimere, Hämoglobin <10 g/dl, Natrium <130 oder >150 mmol/l, Kalium <3 oder >6 mmol/l, Glukose >350 mg/dl, Thrombozyten <100.000/µl, INR <1,2, Kreatinin >2 mg/dl, Lipase >180 U/l, Gesamt/ionisiertes Kalzium <2,2/1,15 oder >2,6/1,35 mmol/l, Bilirubin >1,1 mg/dl, erhöhtes Prokalzitonin
*Relevante Veränderungen in der Blutgasanalyse* Base-Excess <4 oder >4 mmol/l, pH-Veränderung jenseits der Norm, Laktat >2 mmol/l

*ÖGD* Ösophagogastroduodenoskopie, *ERCP* endoskopisch-retrograde Cholangiopankreatikographie, *EKG* Elektrokardiogramm

Die Patientendaten sowie Daten über den klinischen Verlauf wurden aus dem klinischen Informationssystem Medico (Cerner, Idstein) und dem System ERPath (eHealth-Tec, Berlin) abgerufen. Zur statistischen Auswertung der Daten wurde die SPSS-Softwareversion 26 (IBM SPSS Statistics [Armonk, NY, USA]) verwendet. Der Chi-Quadrat-Test wurde für die kategorialen Variablen verwendet. Metrische Variablen wurden als Median und Standardabweichung (SD) dargestellt, wobei der T‑Test verwendet wurde.

Das Studienprotokoll wurde vom institutionellen Ethikrat der Universität Duisburg-Essen genehmigt und in Übereinstimmung mit der Erklärung von Helsinki erarbeitet. Die Studiennummer lautet 19-9060-BO.

## Ergebnisse

Während des Studienzeitraums vom 1. Januar bis zum 31. Dezember 2019 wurden 12.151 Patienten in unserer Notaufnahme aufgenommen und nach dem Manchester Triage System eingeteilt. Von diesen 12.151 Patienten wurden 758 (6,2 %) Patienten in die Dringlichkeitsstufe „rot“, 983 (8,1 %) in die Stufe „orange“, 4275 (35,2 %) in die Stufe „gelb“, 5340 Patienten (44,0 %) in die Dringlichkeitsstufe „grün“ und 795 (6,5 %) in die niedrigste Prioritätsstufe „blau“ eingeteilt.

Diese Studie konzentrierte sich auf die blaue und grüne Dringlichkeitsstufe. Nach Ausschluss von 1595 Patienten (26,0 %; 1434 grün und 161 blau triagierte Patienten), die über den Rettungsdienst aufgenommen wurden, und 502 Patienten (8,2 %; 458 grün und 44 blau triagierte Patienten), die mit der gleichen Beschwerdesymptomatik bereits vorher einen Arzt konsultiert hatten, wurden 3448 grün triagierte Patienten und 590 blau triagierte Patienten in die Studie eingeschlossen. Der Anteil der stationären Aufnahmen war in der blauen Dringlichkeitsstufe signifikant geringer als in der grünen Dringlichkeitsstufe (10,8 % vs. 29,0 %; *p* < 0,001; Tab. 2).

**Table 2:** 

**Dringlichkeitsstufe**	**Grün (*n*** **=** **3448)**	**Blau (*n*** **=** **590)**	**Gesamt (*n*** **=** **4038)**	***p*-Wert**
Ambulante Behandlung; *n* (%)	2220 (64,4)	451 (76,4)	2671 (66,1)	<0,001
Stationäre Aufnahme; *n* (%)	1000 (29,0)	64 (10,8)	1064 (26,4)	<0,001
Ungesehen gegangen; *n* (%)	88 (2,6)	49 (8,3)	137 (3,4)	<0,001
Entlassung gegen ärztlichen Rat (%)	140 (4,1)	26 (4,4)	166 (4,1)	0,695
**Anteil der stationären Aufnahmen einer neuen Untergruppe (CEReCo-blue)**				
**CEReCo blue-Gruppe**	***n*** **=** **340**			
Ambulante Behandlung; *n* (%)	337 (99,1)			
Stationäre Aufnahme; *n* (%)	3 (0,9)			

In der Dringlichkeitsstufe „grün“ waren signifikant mehr männliche (47,6 % vs. 42,5 %; *p* = 0,023) und ältere Patienten (Median 47 Jahre vs. 42 Jahre; *p* < 0,001). Es traten 21 der möglichen 52 Beschwerdebilder der deutschen Version des Manchester Triage Systems in beiden Gruppen auf. Patienten mit pädiatrischen, neurologischen und chirurgischen Beschwerdebildern wurden in der vorliegenden Studie ausgeschlossen. In der blauen Dringlichkeitsstufe gab es einen signifikant geringeren Anteil der Beschwerdebilder Bauchschmerzen, urologisches Problem, Durchfälle und Erbrechen, Allergien, Dyspnoe, Kollaps und einen statistisch signifikant höheren Anteil der Beschwerdebilder psychiatrische Störungen, Unwohlsein bei Erwachsenen und auffälliges Verhalten. Das am häufigsten verwendete Beschwerdebild in beiden Gruppen war Unwohlsein bei Erwachsenen (Tab. 3).

**Table 3:** 

Präsentationsdiagramm: *n* (%)	Grün (*n* = 3448)	Blau (*n* = 590)	Gesamt (*n* = 4038)	*p*-Wert
*Unwohlsein bei Erwachsenen*	1488 (43,2)	345 (58,5)	1833 (45,4)	<0,001
*Abdominelle Schmerzen*	730 (21,2)	94 (15,9)	824 (20,4)	0,004
*Extremitätenprobleme*	265 (7,7)	40 (6,8)	305 (7,6)	0,442
*Urologisches Problem*	250 (7,3)	27 (4,6)	277 (6,9)	0,018
*Atemproblem bei Erwachsenen*	244 (7,1)	23 (3,9)	267 (6,6)	0,004
*Durchfälle und Erbrechen*	139 (4,0)	11 (1,9)	150 (3,7)	0,010
*Thoraxschmerz*	107 (3,1)	19 (3,2)	126 (3,1)	0,880
*Allergie*	58 (1,7)	3 (0,5)	61 (1,5)	0,031
*Kollaps*	47 (1,4)	2 (0,3)	49 (1,2)	0,036
*Gastrointestinale Blutung*	19 (0,6)	6 (1,0)	25 (0,6)	0,182
*Rückenschmerz*	17 (0,5)	6 (1,0)	23 (0,6)	0,118
*Überdosierung und Vergiftung*	21 (0,6)	0 (0)	21 (0,5)	0,057
*Diabetes*	13 (0,4)	1 (0,2)	14 (0,4)	0,428
*Auffälliges Verhalten*	7 (0,2)	5 (0,8)	12 (0,3)	0,008
*Betrunkener Eindruck*	12 (0,3)	0 (0)	12 (0,3)	0,151
*Halsschmerz*	9 (0,3)	3 (0,5)	12 (0,3)	0,308
*Stürze*	7 (0,2)	2 (0,3)	9 (0,2)	0,518
*Asthma*	8 (0,2)	0 (0)	8 (0,2)	0,242
*Vigilanzminderung (UME)*	5 (0,1)	0 (0)	5 (0,1)	0,355
*Psychiatrische Erkrankung*	0 (0)	3 (0,5)	3 (0,1)	<0,001
*Nackenschmerz*	2 (0,1)	0 (0)	2 (<0,1)	0,558
**Total**	**3448 (100)**	**590 (100)**	**4038 (100)**	–

**Figure 1:**
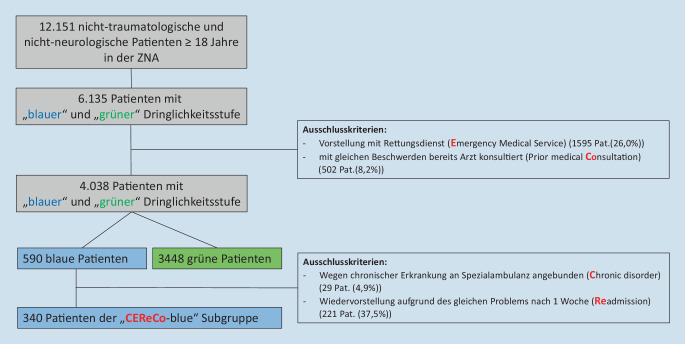

Nach genauerer Betrachtung der Patienten der blauen Dringlichkeitsstufe zeigte sich, dass die zwei Merkmale, Wiedervorstellung aufgrund des gleichen medizinischen Problems innerhalb einer Woche nach stationärer Entlassung, einschließlich postinterventioneller Komplikationen, und das Merkmal chronische Erkrankung mit Anbindung an eine unserer Ambulanzen, besonders häufig vorkamen (entsprechend 221 Patienten [37,5 %] und 29 Patienten [4,9 %]), sodass wir eine Untergruppe der blauen Dringlichkeitsstufe definierten, bei der Patienten mit diesen Merkmalen ausgeschlossen wurden. Diese Untergruppe wurde anhand dieser und der oben erwähnten Ausschlusskriterien, Patienten mit chronischer Erkrankung (**C**hronic disorder), durch den Rettungsdienst aufgenommen (**E**mergency Medical Service), Wiedervorstellung aufgrund des gleichen medizinischen Problems innerhalb einer Woche nach stationärer Entlassung (**Re**admission), und mit der gleichen Beschwerdesymptomatik bereits vorher einen Arzt konsultiert (Prior Medical **Co**nsultation), „CEReCo-blue-Gruppe“ genannt (Abb. 1). Dieser CEReCo-blue-Gruppe konnten nun noch 340 Patienten zugeordnet werden, von denen 3 Patienten (0,9 %) nach unseren Kriterien für eine „gerechtfertigte stationäre Aufnahme“ aufgenommen wurden. Der Verlauf der 3 Patienten wird in Tab. 4 beschrieben. Der Anteil dieser „CEReCo-blue-Patienten“ von allen ursprünglich in der Notaufnahme vorstelligen Patienten beträgt 2,8 %.

**Table 4:** 

**Patient 1**	
*Aktuelle Anamnese*	75-jährige Patientin mit progredienten Sturzneigung bei bekannter Alzheimer-Demenz, Zustand nach pertrochantärer Femurfraktur und operativer Versorgung mit anschließender stationärer Rehabilitation 10 Wochen zuvor
*Vorerkrankungen*	Arterielle Hypertonie, nicht insulinpflichtiger Diabetes mellitus Typ 2, eine monoklonale Gammopathie vom Typ IgG, eine Hyperlipoproteinämie, Mammakarzinom (unter antiöstrogener Therapie)
*Untersuchungsbefund*	Temperatur 37,5 °C, Blutdruck 112/66 mm Hg, Herzfrequenz 100/min, Sauerstoffsättigung unter Raumluft 95 %. Körperliche Untersuchung ohne wesentliche Auffälligkeiten, Operationswunde reizlos
*Elektrokardiogramm*	Normofrequenter Sinusrhythmus mit bekanntem Linksschenkelblock ohne akute Ischämiezeichen oder höhergradige Herzrhythmusstörungen
*Laborchemische Auffälligkeiten*	D‑Dimere 3,6 mg/l (Norm <0,55 mg/l), Leukozyten 12,89/nl (Norm 3,6–9,2/nl), CRP 16,8 mg/dl (Norm <0,5 mg/dl), LDH 304 U/l (Norm 120–247 U/l), GGT 365 U/l (Norm <35 U/l), alkalische Phosphatase 455 U/l (Norm 25–100 U/l)
*Urinuntersuchung*	Unauffällig
*Echokardiographie*	Mittelgradig eingeschränkte linksventrikuläre Pumpfunktion, keine Zeichen der Rechtsherzbelastung
*Ultraschall Abdomen *	Unauffällig
*Röntgenaufnahme des Thorax*	Pneumonisches Infiltrat im rechten Mittellappen
*Bemerkung*	Keine Zeichen einer Thrombose, keine Zeichen einer Lungenembolie. D‑Dimere wurden im Rahmen des entzündlichen Geschehens gewertet
*Aufnahmeindikation*	Verdacht auf Pneumonie, aufgrund des fortgeschrittenen Alters der Patientin erfolgte die stationäre Aufnahme zur antibiotischen und symptomatischen Therapie
**Patient 2**	
*Aktuelle Anamnese*	78-jähriger Patient, seit über einer Woche Appetitlosigkeit und allgemeines Schwächegefühl
*Vorerkrankungen*	Zustand nach Apoplex, arterielle Hypertonie, beinbetontes chronisches Schmerzsyndrom
*Untersuchungsbefund*	Temperatur 36,8 °C, Blutdruck 160/90 mm Hg, Herzfrequenz 90/min, Sauerstoffsättigung unter Raumluft 97 %. Körperliche Untersuchung ohne wesentliche Auffälligkeiten
*EKG*	Normofrequenter Sinusrhythmus ohne akute Ischämiezeichen oder Herzrhythmusstörungen
*Laborchemische Auffälligkeiten*	Laktat 2,3 mmol/l (Norm 0,5–1,6 mmol/l), Leukozyten 10,05/nl, (Norm 3,6–9,2/nl), Prokalzitonin 0,79 ng/ml (Norm <0,5 ng/ml), GGT 716 U/l (Norm <55 U/l), GOT 227 U/l (Norm <50 U/l), GPT 165 U/l (Norm <50 U/l), Bilirubin (gesamt) 1,5 mg/dl (Norm 0,3–1,2 mg/dl), Bilirubin (direkt) 0,7 mg/dl (Norm <0,2 mg/dl), alkalische Phosphatase 283 U/l (Norm 25–124 U/l), Lipase 99 U/l, (Norm 5,6–51,3 U/l), LDH 421 U/l (Norm 100–247 U/l), Kreatinin 2,23 mg/dl (Norm 0,9–1,3 mg/dl)
*Aufnahmeindikation*	Verdacht auf Cholezystitis
**Patient 3**	
*Aktuelle Anamnese*	20-jährige Patientin, seit zwei Wochen bestehende Unterbauchschmerzen mit einer Periodenblutung von wechselnder Intensität
*Vorerkrankungen*	Zustand nach Appendizitis mit konsekutiver Appendektomie Untersuchungsbefund: Temperatur 37,0 °C, Blutdruckwert 110/64 mm Hg, Herzfrequenz 100/min, Sauerstoffsättigung unter Raumluft 98 %. In der körperlichen Untersuchung diffuser abdomineller Druckschmerz ohne Abwehrspannung und ohne Resistenzen bei regelrechten Darmgeräuschen
*Laborchemische Auffälligkeiten*	β‑hCG 917,6 mlU/ml (Norm <6 mlU/ml), D‑Dimere 2,76 mg/l (Norm <0,55 mg/l), Leukozytose 12,82/nl (Norm <9,2/nl) hypochrome, mikrozytäre Anämie mit Hb 9,3 g/dl (Norm 12–15,2 g/dl)
*Ultraschall Abdomen*	Vergrößerter Uterus, Raumforderung im Bereich des rechten Ovars, freie Flüssigkeit im Unterbauch
*Aufnahmeindikation*	Verdacht auf Extrauteringravidität, nach gynäkologischer Mitbeurteilung Indikation zur Notfalloperation am selben Tag

## Diskussion

Diese Studie untersucht die Notfallpatienten, die in der Manchester Triage der niedrigsten Prioritätengruppe „blau“ zugeordnet sind. Es gilt herauszufinden, ob Patienten, die in der Triage der Kategorie blau zugeteilt werden, nachdem sie sich selbst in der Notaufnahme vorgestellt haben, unmittelbar an einem gemeinsamen Tresen in integrierten Notfallzentren (INZ) an die ambulante Versorgung weitergeleitet werden können, um die Notaufnahmen zu entlasten und nicht unnötig Ressourcen zu binden.

In einigen Ländern, eben auch in Deutschland, wird die Notfallversorgung re-strukturiert, um die Akut- und Notfallversorgung zu optimieren [5]. Daher wird ein zuverlässiges Triage-System benötigt, um bereits frühzeitig zwischen ambulanter und Krankenhauszuweisung zu differenzieren [11]. Verschiedene Untersuchungen belegen bereits eine niedrige 30-Tage-Mortalität bei niedrig prioritären Notfallpatienten [3, 13, 15]. Slagman et al. [12] analysierten in einer prospektiven Studie die Manchester Triage als „Tool“, um Patienten unmittelbar dem ambulanten Sektor (hausärztliche Versorgung, ambulante KV-Praxis) zuzuweisen. Dabei wurden „grün“ und „blau“ triagierte Patienten (MTS) als nicht dringlich kategorisiert und bezüglich der Mortalität untersucht. Hierbei zeigte sich eine stationäre Aufnahmerate bei den „nicht dringlichen“ Patienten von immerhin 29,6 % und kein signifikanter Unterschied bezüglich der Kurz- und Langzeit-Mortalität zwischen der nicht dringlichen (blau und grün) und der dringlichen (rot, orange und gelb) kategorisierten Patientengruppe, sodass von dieser Arbeitsgruppe die Anwendung des MTS zur Selektion von Patienten mit ambulanter Behandlungsindikation nicht empfohlen wurde.

Ziel dieser Untersuchung war eine Gruppe zu finden, die mithilfe des MTS ohne Risiken und Schaden für den Patienten zur ambulanten Versorgung weitergeleitet werden kann, um die Notaufnahmen zu entlasten. Wir nahmen an, dass die Kategorie „blau“ primär diese Patienten beinhaltet. Daher haben wir Patienten der Kategorie „blau“ zunächst mit denen der Kategorie „grün“ verglichen und schlossen dabei alle Patienten aus, die nach vorheriger ärztlicher Konsultation mit Einweisung oder mit dem Rettungsdienst vorstellig wurden. Obwohl sich ein signifikanter Unterschied in der Krankenhausaufnahme zwischen grün und blau zeigte (10,8 % vs. 29,0 %, *p* < 0,001), die Patienten der Kategorie grün wurden häufiger stationär aufgenommen, war die Anzahl der blau triagierten und stationär aufgenommenen Patienten immer noch zu hoch, um diese ohne Schaden für die Patienten unmittelbar der ambulanten Versorgung zuzuweisen.

In Deutschland obliegt die Aufnahmeentscheidung dem behandelnden Notaufnahmearzt und ist abhängig von Diagnostik, Komorbiditäten und natürlich dem Zustand des Patienten [10]. Wir definierten daher weiterhin strikte Aufnahmekriterien (Tab. 1). Auf der Suche nach einer alternativen Möglichkeit innerhalb der MTS als Tool, um Patienten nach der Triagierung zwischen Notaufnahme und ambulanter Weiterversorung zu selektieren, definierten wir innerhalb der blauen Kategorie eine Subgruppe. Wir untersuchten, welche Charakteristika die blauen, stationär aufgenommenen Patienten gemeinsam aufwiesen. Wir konnten zeigen, dass eine Wiedervorstellung binnen einer Woche nach Krankenhausaufenthalt wegen derselben Diagnose, eine chronische Erkrankung, wegen der die Patienten an eine Spezialambulanz unserer Universitätsmedizin angebunden waren, überproportional häufig zu stationären Aufnahmen führten.

Mit der Definition von diesen weiteren Ausschlusskriterien konnten wir die „CEReCo-blue-Gruppe“ benennen, die neben den o. g. Kriterien (**C**hronic Disorder, **Re**admission) die bis dato festgelegten Ausschlusskriterien (**E**mergency Medical Service und Prior Medical **C**onsultation) beinhaltete. Dies führte dazu, dass sich innerhalb der blau triagierten Patienten nur noch eine „gerechtfertigte“ Aufnahmerate von 0,9 % zeigte. Die festgelegten Ausschlusskriterien stellen sich wie folgt dar:

Chronische Erkrankung (Chronic Disorder): Ausgeschlossen wurden Patienten, die wegen einer chronischen Grunderkrankung an unsere Klinik angebunden sind. Dabei handelt es sich um onkologische, organtransplantierte und Dialyse-Patienten sowie Patienten, die an chronischen infektiösen Erkrankungen leiden, wie HIV oder Hepatitis. Die Komplexität dieser Erkrankungen übersteigt oft das Niedergelassenen-Setting und macht eine Krankenhausaufnahme häufiger notwendig. Eine Studie von Seiger et al. bei pädiatrischen Patienten unter 16 Jahren zeigte, dass die Sensitivität des MTS bei chronisch Erkrankten niedriger ist [11]. In unserer Studie traf das Ausschlusskriterium chronische Erkrankung auf 4,9 % der blau triagierten Patienten zu.

Rettungsdienstzuweisung (Emergency Medical Service): Da der Rettungsdienst primär für die Zuweisung von akut erkrankten Patienten zuständig ist, wurden diese Patienten ausgeschlossen. Von den blau triagierten Patienten wurden 16,5 % mit dem Rettungsdienst in unsere Notaufnahme gebracht.

Wiedervorstellung: Patienten, die sich binnen einer Woche erneut nach Krankenhausentlassung wegen derselben Problematik vorstellten, wurden ebenfalls ausgeschlossen, in der Annahme, dass die stationäre Behandlung somit nicht ausreichend gewesen und wieder notwendig ist. Daher wurden Patienten, die sich mit Komplikationen nach Interventionen, wie Herzkatheteruntersuchungen, endoskopischen Eingriffen oder Operationen vorstellten, ebenfalls ausgeschlossen. Hierdurch wurden in unserer Studie 37,5 % der blau triagierten Patienten ausgeschlossen.

Facharzteinweisung oder nach vorheriger ärztlicher Konsultation: Patienten, die bereits wegen derselben Beschwerden Arztkontakt hatten oder bestenfalls fachärztlich eingewiesen wurden, schlossen wir dahingehend aus, dass somit entweder die ambulante Therapie nicht ausreichend oder die stationäre Aufnahme bereits aus ärztlicher Sicht notwendig war. Aufgrund dieses Ausschlusskriteriums wurden in dieser Studie 8,2 % der blau triagierten Patienten nicht der CEReCo-blue-Gruppe zugeteilt.

Schätzungen gehen von einer Aufnahmequote von 13–16 % bei grün und blau triagierten Patienten aus [3, 6, 13, 15]. Slagman et al. [12] konnten eine Aufnahmerate von 29,6 % zeigen. Unsere Aufnahmequote lag vor Definition der Kriterien bei 26,4 %. Allerdings konnten wir nach Definition der „CEReCo-blue-Gruppe“ eine deutliche Reduktion der „gerechtfertigten stationären Aufnahmen“ zeigen. Nur 3 Patienten (0,9 %) wurden innerhalb dieser Gruppe stationär aufgenommen.

Die drei Patienten, die in der CEReCo-blue-Gruppe die Kriterien für eine gerechtfertigte stationäre Aufnahme erfüllten, sind in Tab. 4 zusammengefasst. Auch wenn Patient 1 und 2 gerechtfertigt stationär aufgenommen wurden, wäre eine zumindest vorerst ambulante Behandlung mit einer oralen antibiotischen Therapie bei beiden Patienten möglich gewesen, ohne diese Patienten zu gefährden. Bei Patientin 3 war eine innerklinische Notfallversorgung zweifelsfrei indiziert, und diese Patientin wäre klar gefährdet gewesen, wenn sie einer ambulanten Weiterbehandlung zugeteilt worden wäre. Es wäre vielleicht zu diskutieren, ob eine Anwendung der CEReCo-blue-Gruppe dadurch ergänzt werden müsste, dass man bei weiblichen Patienten mit abdominellen Beschwerden im gebärfähigen Alter einen βHCG-Test im Urin durchführen lässt, bevor man diese Patienten der ambulanten Weiterversorgung zuteilt.

Die aktuellen nationalen Diskussionen über die grundsätzlichen Möglichkeiten und Grenzen von Triage-Systemen zur Patientensteuerung zwischen ambulanter kassenärztlicher und in der Notaufnahme durchgeführter Notfallversorgung belegen den Wert von Untersuchungen wie dieser Studie. Wir konnten mit einfach definierten Kriterien und Einführen der CEReCo-blue-Gruppe ein vielversprechendes Tool aufzeigen, welches in dieser Studie eine mit 0,9 % niedrige Fehlerquote zeigt. Allerdings schloss diese CEReCo-blue-Gruppe in der Notaufnahme unseres Uniklinikums lediglich einen Anteil von 2,8 % aller Patienten ein, wohingegen die ganz überwiegende Mehrzahl mit mehr als 97 % tatsächlich Leistungsmerkmale der Notaufnahme erforderten. Nach unserem Wissen ist dieses die erste Studie, die schrittweise ein Patientenkollektiv des Manchester Triage Systems selektiert, um Patienten, ohne sie zu gefährden, der ambulanten Versorgungsebene zuzuteilen. Derzeit ist kein wissenschaftlich validiertes Ersteinschätzungssystem bekannt, dass diese Aufgabe erfüllt. Im Jahr 2017 wurde entschieden, ein Ersteinschätzungsverfahren in Deutschland (Strukturierte medizinische Ersteinschätzung, SmED) auf Grundlage des Swiss Medical Assessment Systems (SMASS) zu entwickeln [8]. Dieses nichtvalidierte System soll zur flächendeckenden telefonischen Ersteinschätzung implementiert werden, und es wird diskutiert, ob es mit entsprechenden Modifikationen auch die Ersteinschätzung an einem gemeinsamen Tresen in den Notaufnahmen unterstützen könnte. Da validierte Ersteinschätzungssysteme in den Notaufnahmen bereits angewendet werden, halten die Autoren dieser Studie es für naheliegend, ein in Deutschland bereits etabliertes System für den Tresen der Notaufnahmen zu verwenden. Am häufigsten wird in Deutschland das Manchester Triage System verwendet, sodass die Anwendung einer Modifikation dieses Systems mit dem Ziel, Patienten in der Notaufnahme einer ambulanten Versorgungsebene zuteilen zu können, einleuchtend erscheint und unserer Meinung nach die bessere Alternative zum nichtvalidierten SmED-System darstellt. Ob sich der Anteil der CEReCo-blue-Gruppe in anderen Notaufnahmen ähnlich darstellt, wäre zu untersuchen. Aktuell wäre die Entlastung der Notaufnahmen durch die CEReCo-blue-Gruppe relativ gering (2,8 % am Gesamtaufkommen der Notfallpatienten). Die Ergebnisse dieser Studie beruhen auf Untersuchungen von Patienten mit primär nichttraumatologischen und primär nichtneurologischen Beschwerden. Ergänzende Studien mit traumatologischen und neurologischen Patienten sind daher sinnvoll. Nach Meinung der Autoren ist es gut vorstellbar, dass sich die Anzahl der Patienten einer äquivalenten Patientensubgruppe von Patienten mit primär traumatologischen und primär neurologischen Beschwerden deutlich erhöht und somit zu einer wesentlichen Entlastung der Notaufnahmen beitragen könnte. Außerdem zeigt unsere Untersuchung auf, dass eine Entlastung der Notaufnahmen durch Schärfung der Triagekriterien innerhalb eines etablierten Systems möglich ist und deshalb auf dem Boden eines solchen Systems aufbauen sollte. Der sehr hohe Anteil an nicht risikoarm in kassenärztlichen Strukturen zu versorgenden Notfallpatienten in unserer Untersuchung weist darauf hin, dass eine räumliche oder organisatorische Trennung von ambulanter und stationärer Notfall- und Akutversorgung in voneinander getrennte Systeme nicht sinnvoll erscheint.

## Studienlimitierung

Diese Studie ist retrospektiv und somit sollten einige Einschränkungen in Betracht gezogen werden. Diese Studie beinhaltet nichttraumatologische Notfallpatienten. Außerdem wurden Patienten <18 Jahren sowie unfallchirurgische und neurologische Patienten ausgeschlossen.

## Fazit für die Praxis

Verglichen wurden Patienten, die mittels MTS der Kategorie blau zugeordnet wurden mit Patienten der nächsthöheren Dringlichkeitsstufe grün mit der Fragestellung, welche Patienten der ambulanten Weiterversorgung zugewiesen werden können. Obwohl die Anzahl der stationären Aufnahmen in der blauen Gruppe deutlich seltener vorkam als in der grün triagierten Gruppe, war sie mit 10,8 % relativ hoch. Nachdem weitere Ausschlusskriterien, wie chronische Erkrankung, Rettungsdienstzuweisung, Wiedervorstellung nach stationärem Aufenthalt, angewendet wurden (CEReCo-blue-Subgruppe innerhalb der Kategorie blau), konnten wir deutlich die Anzahl der stationären Aufnahmen senken. Wir schlagen daher diese weitere Gruppierung innerhalb der MTS als sinnvolles Tool vor, um Patienten ohne Arztkontakt perspektivisch an Tresen von integrierten Notfallzentren der primär ambulanten Notfallversorgung zuzuweisen. Der sehr hohe Teil an Notfallpatienten mit Erfordernis von Leistungen der Notaufnahme auch bei im Ergebnis ambulanter Behandlung betont die Wichtigkeit integrierter Strukturen von Notfallzentren ohne räumliche oder organisatorische Trennung.
